# Regional Epidemiological Study on the Dental Status of the First Permanent Molar in Romanian Schoolchildren

**DOI:** 10.3390/dj13010026

**Published:** 2025-01-10

**Authors:** Liana Beresescu, Gabriela Felicia Beresescu, Daniela Esian, Alexandru Vlasa, Csilla Benedek, Raluca Sabau, Alexandra Mihaela Stoica

**Affiliations:** 1 Faculty of Dental Medicine, George Emil Palade University of Medicine, Pharmacy, Science, and Technology of Târgu-Mureș, 540139 Târgu-Mureș, Romania; liana.beresescu@umfst.ro (L.B.); daniela.esian@umfst.ro (D.E.); alexandru.vlasa@umfst.ro (A.V.); csilla.benedek@umfst.ro (C.B.); alexandra.stoica@umfst.ro (A.M.S.); 2 Private Practice, Dentral, Moldova St., no. 22, 540542 Târgu-Mureș, Romania; raluca.sabau@yahoo.com

**Keywords:** permanent first molar, dental caries, ICDAS II classification, sealings, fillings

## Abstract

**Background/Objectives:** Dental caries remains a significant public health challenge in Romania, with recent studies reporting a prevalence of 40% in children’s permanent teeth, with 90% of cases untreated. This study aimed to evaluate the dental status of the first permanent molars in children aged 11–12 years. **Methods:** This cross-sectional study was conducted over 12 months at the Integrated Center for Dental Medicine in Târgu Mureș and two private clinics in Transylvania. A total of 516 children, aged 11–12 years, were examined using the ICDAS II classification. Data on carious lesions and dental treatments performed were collected. **Results:** Of the 2064 first permanent molars examined, 57.99% had carious lesions, fillings, or extractions, while 41.28% were free from caries. Among the affected molars, 41.71% had untreated caries, 9.30% were filled, and 6.25% were sealed. Boys showed a significantly higher prevalence of advanced lesions (ICDAS 4–6) compared to girls. Caries predominantly affected the pits and fissures (87.46%). **Conclusions:** This study reveals a high prevalence of carious lesions in first permanent molars and a low rate of treatment. The findings emphasize the need for improved oral health education, increased access to dental care, and the development of national strategies to prevent and treat dental caries in children.

## 1. Introduction

According to the World Health Organization (WHO), dental caries remains one of the most prevalent diseases globally, affecting a significant proportion of the child population. The primary contributing factors to this issue are the high consumption of sugars, poor oral hygiene, and the insufficient use of fluoride [1].

The first permanent molar, known as the “six-year molar”, plays a crucial role in the development of the permanent dentition. It erupts around the age of six and significantly impacts occlusion, the coordination of masticatory forces, and the maintenance of a balanced dental arch. Six-year molars contribute to the correct alignment of the teeth and the development of the maxillary and mandibular structure, being essential to overall oral function. They also serve as a reference point in the transition from primary to permanent dentition [2,3].

The eruption of the six-year molar is not only a functional step but also a critical stage in the development of occlusion. By stabilizing both anterior and posterior tooth movements, it contributes to the increase in occlusal height. As the first permanent tooth to erupt, the six-year molar acquires a central role in mandibular kinetics and supports occlusal function until the eruption of the incisors and canines [4,5]. The early loss of, or significant damage to, this molar can affect overall dental functionality, requiring complex orthodontic and restorative interventions [6,7,8].

The six-year molar exhibits specific morphological features that mean that it is predisposed to the development of caries. Its occlusal surface has deep grooves and fossae, and the development of adjacent spaces promotes the formation of retention points for bacterial plaque. These characteristics make it vulnerable to caries, especially during the early stages of dental eruption [9,10]. Eruption, which can last for more than a year, contributes to this vulnerability, given that self-cleaning is insufficient and the enamel is less mineralized during the post-eruptive period [11]. These aspects emphasize the importance of proper oral hygiene and adequate dietary habits during this critical stage of development [12].

In Romania, the prevalence of carious lesions in children remains alarmingly high, affecting both urban and rural populations. However, socio-economic disparities exacerbate the issue, with children from economically disadvantaged areas facing additional challenges due to inadequate oral health education, limited access to dental care, and the underutilization of preventive measures, such as dental sealants. The absence of national oral health programs further increases the risk of untreated carious lesions and their complications across all socio-economic groups, with vulnerable populations being disproportionately impacted [13,14].

The social and economic costs of untreated dental caries are significant. Carious lesions in children contribute not only to oral health complications but also to reduced school performance due to pain and discomfort, increased absenteeism, and higher healthcare expenditures for invasive treatments [15,16]. Targeted public health interventions, including educational programs and improved access to preventive services, could significantly reduce the prevalence of caries and the long-term burden of oral disease.

Considering its essential role in the development of permanent dentition, the six-year molar can be regarded as an important indicator of the overall health of the oral cavity [17,18]. The health of this tooth not only reflects the current condition of the oral cavity but also influences the future evolution of permanent dentition. Although numerous studies have explored the health of the six-year molar, there is still a significant need for knowledge regarding its dental status and early prevention and intervention strategies to address practical challenges. This study differentiates itself from previous research by focusing specifically on the dental status of the six-year molar in the population of Romania, considering the socio-economic and educational particularities of this country, which can significantly influence the dental health of children. Therefore, this research contributes important insights, highlighting the local challenges and needs regarding the prevention and treatment of dental caries in this crucial tooth. The critical role of the six-year molar underscores the importance of continuous monitoring and preventive interventions throughout the eruption and maturation of this tooth, given its impact on long-term general dental health [5].

Globally, the six-year molar presents a significant risk for caries, and recent studies suggest a high prevalence of carious lesions, especially among children in low- and middle-income countries [19,20,21]. In Romania, the health of the first permanent molar is a major concern, influenced by factors such as insufficient education regarding oral hygiene, a high consumption of cariogenic foods, and limited access to preventive dental services. Socio-economic disparities and the lack of effective prevention programs contribute to the high prevalence of carious lesions, emphasizing the urgent need for educational interventions, improved accessibility to dental treatments, and comprehensive national strategies to address these systemic challenges [13,14,22,23].

The aim of this study was to evaluate the dental status of the first permanent molar at the time of the completion of permanent dentition eruption in a cohort of children aged between 11 and 12 years, to identify the prevalence of carious lesions and the dental interventions performed.

This study aimed to address a critical gap in the literature by providing comprehensive data on the dental status of first permanent molars among Romanian schoolchildren, a population that has been underrepresented in global oral health studies. By focusing on a regional cohort, the research sheds light on specific socio-economic, educational, and systemic challenges that influence dental health outcomes. These findings contribute to the global understanding of oral health disparities and inform targeted interventions for similar populations in other countries with comparable socio-economic conditions.

Based on the study’s objectives, the following null hypotheses were proposed:

- H_01_: There is no significant difference in the prevalence of carious lesions in the first permanent molar between boys and girls.
- H_02_: There is no significant difference between the prevalence of untreated carious lesions and treated carious lesions (fillings, sealants, and extractions) in the first permanent molar.

This study not only highlights the dental health challenges faced by Romanian schoolchildren but also underscores the need for public health strategies addressing socio-economic vulnerabilities, accessibility to care, and the societal costs of untreated dental disease. By bridging these gaps, the research provides valuable insights to inform targeted interventions and improve oral health outcomes in similar populations.

## 2. Materials and Methods

The study was conducted over a 12-month period at the Integrated Center for Dental Medicine of the University of Medicine, Pharmacy, Science and Technology “George Emil Palade” in Târgu Mureș and at two private clinics in two cities in Transylvania (Alba Iulia and Bistrița Năsăud). Ethical approval was obtained from the Ethics Committee of the University of Medicine, Pharmacy, Science, and Technology “George Emil Palade” in Târgu Mureș (approval no. 2064, date of approval: 9 February 2023).

The patients were selected from schools in these cities with the help of the school administration. The selection criterion was as follows: cooperative patients aged between 11 and 12 years. Children who were non-cooperative or whose parents (legal guardians) did not consent to their participation in the study were excluded. Prior to the examination, the parents or legal guardians of the children were informed about the study protocol, and informed consent was obtained.

All examinations were conducted by three experienced dentists. Before the start of data collection, the three participating practitioners underwent a comprehensive training and calibration process to ensure consistency and accuracy in their evaluations. This training involved a dedicated calibration course based on the International Caries Detection and Assessment System (ICDAS II) criteria [24]. The course was conducted over a two-week period and consisted of both theoretical and practical components. The theoretical component included detailed presentations and discussions on the ICDAS II classification system, including the diagnostic criteria for carious lesions at different stages of severity. Participants were provided with a manual of ICDAS II guidelines and diagnostic criteria to study. The practical component involved hands-on training using clinical photographs and typodont models with artificially created carious lesions representative of the ICDAS II codes (0–6). Participants practiced classifying lesions under guided supervision and received immediate feedback on their accuracy. Periodic recalibration sessions during the study maintained examiner reliability. The dental examination was performed under standard conditions offered by the dental unit, both visually and tactilely on clean, moist, and dry teeth, according to the ICDAS II classification recommendations. A blunt-ended probe was used to remove bacterial plaque and assess the status of the occlusal surface, identify carious lesions, and check for the presence of sealants and fillings.

The ICDAS II classification used to assess the six-year molar is organized in the following table (Table 1):

All participants received detailed instructions on correct oral hygiene, based on the Bass brushing technique or the proper use of electric toothbrushes, and were educated on healthy eating habits, focusing on reducing sugar consumption and promoting healthy snacks. The instruction was carried out using demonstrative models provided by the Discipline of Preventive and Community Dentistry at the Faculty of Dentistry.

### 2.1. Sample Size Determination

The sample size calculation was performed using an estimated prevalence of 50% (a conservative assumption to maximize the sample size), a confidence level of 95%, and a margin of error of 5%, which resulted in a minimum required sample size of 384 participants. To ensure robust data and account for potential data loss, the final sample size was increased to 516 participants.

To further substantiate the adequacy of our sample size, we performed a post hoc power analysis using the observed prevalence of carious lesions in the study (57.99%). With this sample size and the prevalence rate, the study achieved a statistical power of over 90% to detect significant differences in the caries prevalence across various groups, such as gender and ICDAS II classifications. This ensured that our findings were both statistically robust and generalizable to the target population of Romanian schoolchildren aged 11–12 years.

### 2.2. Statistical Analysis

Statistical analysis was performed using GraphPad Prism™ V6.01 software. To assess significant differences between groups, the Chi-square (X^2^) test was applied for categorical data. This method was used to compare the observed frequencies of decayed, sealed, filled, and extracted molars based on various categories such as sex, city, and location on the maxilla or mandible. The *p*-values obtained were used to determine the statistical significance of the observed differences, considering a significance threshold of *p* ≤ 0.05.

To ensure inter-examiner reliability, all three dentists independently examined and classified a set of 50 anonymized cases under identical conditions. The results were compared, and a kappa coefficient was calculated to assess agreement, which exceeded 0.90, indicating excellent reliability.

## 3. Results

The study included 516 children, with a mean age of 11.7 years (SD = 0.6), consisting of 294 girls and 222 boys, resulting in a total of 2064 permanent first molars examined. Of these, 57.99% exhibited carious lesions, fillings, or extractions, while 41.28% were free from caries. Untreated carious lesions were observed in 41.71% of molars, while 9.30% had fillings, and 6.25% were sealed. A total of 0.73% were extracted due to severe caries. The dental status of the examined first permanent molars is presented in the following table (Table 2).

The distribution of carious lesions revealed that 87.46% were located on occlusal surfaces or associated with vestibular and palatal pits. Proximal lesions accounted for 8.16%, while atypical lesions and roots represented 4.36%. (Table 3).

No significant differences were observed between maxillary and mandibular molars regarding the prevalence of carious lesions *p* > 0.05). When comparing data by sex, no significant differences were observed regarding the number of sealed or filled molars, except for ICDAS classification II, codes 3 and 4–6. This showed that boys had a significantly higher prevalence of advanced carious lesions (ICDAS 4–6) compared to girls (36.83% versus 28.83%) (*p* = 0.031), while girls had more frequent stage 3 lesions (20.66% versus 10.81%) (*p* = 0.025). Statistical analysis confirmed a significant difference between untreated and treated carious lesions. Untreated caries accounted for 41.71%, while treated lesions (fillings, sealants, and extractions) collectively represented 16.28% (*p* < 0.001).

Testing of the Null Hypotheses:

- Following the data analysis, H_01_ was rejected. The results demonstrated that boys exhibited a significantly higher prevalence of advanced carious lesions (ICDAS 4–6) compared to girls, confirming a gender-based difference in the severity of caries.
- Similarly, H_02_ was rejected. The analysis revealed that untreated carious lesions (41.71%) were significantly more prevalent than treated carious lesions, which collectively accounted for 16.28% (combining fillings, sealants, and extractions).

## 4. Discussion

Our study highlights a concerning prevalence of carious lesions in the first permanent molars, with 57.99% of molars affected by untreated caries, fillings, or extractions, while only 41.28% were identified as free from caries. These figures align with data from other Central and Eastern European countries, where the caries prevalence ranges between 60% and 70% [25,26,27,28,29]. In these countries, national studies have provided a clear picture of oral health and can contribute to the development of effective public policies in dentistry.

In Romania, national studies are limited, with only three such research projects identified in the specialized literature. The first, conducted in the 1990s [30], was a study that included only five major cities, excluding rural areas, and, thus, it is more difficult to generalize its conclusions. Another longitudinal study, conducted between 1992 and 2011 [31], confirmed the high prevalence of carious lesions among children and adolescents, but it too had a limited scope. More recently, between 2019 and 2020, the “Romanian Oral Health Survey” was conducted, assessing the oral health of children in urban and rural areas, confirming the alarming prevalence of dental caries [32]. However, this study also provides limited information and does not fully reflect the national situation. Most of the research in Romania remains regional [33,34,35], resulting in a fragmented picture of oral health and emphasizing the need for comprehensive, updated national studies.

Our findings align with the limited national studies available in Romania, which also report a high prevalence of dental caries among children. For example, the first study conducted in the 1990s revealed a DMFT index ranging from 3.0 to 5.9 in children aged 11–13 years, highlighting the widespread nature of carious lesions even in urban populations [30]. Subsequent studies, such as the longitudinal research conducted between 1992 and 2011, showed a slight improvement, with the DMFT index decreasing to values between 1.5 and 5.6 in the same age group, but still indicated significant oral health challenges [31]. More recent data from the 2019–2020 Romanian Oral Health Survey confirmed the high prevalence of caries, with 40% of children having caries in permanent teeth, of which 90% were untreated, emphasizing the persistent lack of curative and preventive interventions [32]. Our study supports these findings, reporting that 57.99% of first permanent molars were affected by untreated caries, fillings, or extractions, and only 41.28% were caries-free. This detailed focus on the first permanent molar complements the broader trends identified in national studies, emphasizing the urgent need for targeted preventive strategies. Integrating data from regional and national studies into future research will allow a more comprehensive understanding of carious pathology and help identify specific clusters, facilitating the development of effective public health interventions tailored to the needs of Romanian children. For our study, we chose to use the ICDAS II classification to analyze not only the prevalence of carious lesions but also their severity, along with the appropriate treatment options. Carious lesions classified as scores 1 and 2, limited to the enamel and with the potential for remineralization or arrest, were included in the 41.28% of teeth considered free from caries. The highest percentage of carious lesions was found at the level of the pits and fissures (87.46%), the areas most prone to caries. The ICDAS II classification system played a crucial role in guiding both preventive and curative treatment decisions in this study. Lesions classified as scores 1 and 2 were targeted for non-invasive interventions, such as therapeutic sealing, which aligns with evidence suggesting that early-stage lesions can be effectively managed without resorting to invasive procedures, thereby preserving tooth structure and reducing long-term complications [36,37,38]. In contrast, lesions categorized as scores 3–6, indicative of more advanced stages involving dentin or visible cavitation, required curative interventions such as fillings, pulp capping, endodontic treatments, or, in severe cases, extractions. These treatment decisions were guided by lesion severity, the associated risk of pulp involvement, and the need to prevent further disease progression. By stratifying interventions based on lesion severity, the use of ICDAS II ensured that clinical decisions were tailored to the individual needs of each patient, optimizing outcomes and emphasizing a minimally invasive approach whenever possible. This stratified approach not only enhances clinical decision-making but also aligns with the principles of minimally invasive dentistry, ultimately improving long-term oral health outcomes for children.

Preventive strategies remain at the forefront in combating dental caries. Therapeutic sealing, which can effectively manage lesions classified as scores 1 and 2, has been widely supported in the literature as a simple and cost-effective method for halting the progression of incipient caries [36,37,38]. Despite its efficacy, our study revealed that only 6.25% of molars were sealed, reflecting significant systemic barriers to its adoption in Romania, including limited access to dental services and a reluctance among practitioners to adopt sealing as a preventive strategy [39,40,41]. Addressing these challenges is essential in expanding access to preventive care and achieving better oral health outcomes for children.

In addition to sealants, population-level interventions, such as fluoride applications, play a pivotal role in strengthening enamel and reducing caries risk. However, Romania lacks a national water fluoridation program, and natural fluoride levels in drinking water are low, limiting its preventive potential [42]. Bridging this gap through targeted fluoride programs could significantly enhance the efficacy of preventive care. Innovative techniques, such as the Er–YAG laser, also offer promising alternatives for managing early-stage carious lesions [43]. This technology effectively reduces microbial populations while minimizing patient discomfort, making it particularly suitable for uncooperative or vulnerable populations. Integrating such advancements into traditional methods like sealants and fluoride treatments represents a comprehensive approach to improving oral health outcomes among children.

Although the number of extracted molars is low, it raises concerns given the age of the children in the study. The premature loss of these teeth may significantly impact the development of the dental arches, underscoring the importance of timely preventive interventions. In our analysis, the rejection of hypothesis H_02_, which posited no significant differences between untreated and treated carious lesions, highlights critical gaps in the access to, and utilization of, preventive dental care. Similarly, the rejection of hypothesis H_01_, which posited no significant gender-based differences, underscores disparities in the prevalence of advanced lesions. This finding highlights systemic barriers that hinder both preventive and curative interventions, leaving many children at risk of disease progression. Significant gender differences were identified, with boys being more affected. This aligns with previous findings linking poor oral hygiene habits, dietary patterns, and hormonal influences with higher rates of caries among boys [44,45]. These differences highlight the need for gender-targeted interventions, including tailored educational programs and regular screenings.

Systemic challenges, including limited access to dental care and the underutilization of preventive or curative treatments, contribute to the high prevalence of untreated carious lesions in Romanian children. These observations align with reports from other low- and middle-income countries, which document similar trends in the burden of untreated dental conditions [46,47]. Additionally, limited health education programs targeting disadvantaged families contribute to the low awareness of preventive measures, such as regular check-ups and sealants. Cultural perceptions and mistrust in modern dental treatments also play a role in the underutilization of preventive care, as highlighted in studies conducted in Eastern Europe [48,49].

Addressing these systemic barriers requires urgent public health interventions tailored to socio-economic disparities. National programs focused on improving access to affordable preventive and curative care are critical in reducing the prevalence of dental caries. By tackling these challenges, Romania can work toward equitable oral health outcomes for all children, regardless of their economic or geographic background.

In the absence of national prevention and oral health education programs, modern preventive methods, such as dental sealants, are underutilized, and curative interventions are limited. Thus, the results of this study confirm the need for a change in the national oral health strategy. Public education, increased access to preventive treatments, and the implementation of public policies that support oral health are essential in reducing the prevalence of dental caries and improving the dental prognosis of children.

### Limitations of the Study

Our study has several limitations that may affect the generalization of the results. First, its regional focus provides only a partial picture of the nationwide prevalence of carious lesions in the first permanent molars and the overall dental health status in Romania. Additionally, the limited sample size reduces the statistical power of the findings and makes it difficult to extrapolate the conclusions to the entire child population of the country.

This study also focused exclusively on the epidemiological evaluation of the dental status of the first permanent molar, without collecting specific data on risk factors such as diet, fluoride use, toothbrushing habits, compliance with regular check-ups, or the use of orthodontic appliances. While these factors undoubtedly influence the occurrence of caries, analyzing them was beyond the scope of this study.

Furthermore, our study did not include the radiographic examination of interproximal areas, relying solely on the ICDAS II system for visual inspection. While this method is practical for large-scale epidemiological studies and aligns with our objectives, it may limit the accuracy in detecting early-stage carious lesions in interproximal spaces.

Another limitation is the absence of a control group or direct comparison to similar populations in other countries. This restricts our ability to fully contextualize the findings and may limit their broader applicability. However, our results are consistent with those of studies conducted in other low- and middle-income countries, which report similar challenges in oral health outcomes and accessibility to care [50,51].

To address these limitations, future research should aim to include a larger and more diverse sample size, consider the collection of detailed data on risk factors such as diet and oral hygiene habits, incorporate radiographic assessments for greater diagnostic precision, and conduct comparative analyses with populations in different socio-economic and cultural settings to provide a more comprehensive understanding of carious lesions and their determinants.

Nonetheless, the findings have broader implications. Challenges such as limited access to preventive care, socio-economic disparities, and the underutilization of preventive treatments like sealants are not unique to Romania and may reflect similar issues in other low- and middle-income countries. However, caution is advised when generalizing these results to other populations, as cultural, economic, and healthcare system differences may influence outcomes.

## 5. Conclusions

Our study highlighted a high prevalence of carious lesions in the first permanent molars, correlated with a very low number of preventive and curative treatments. These results suggest an unfavorable long-term prognosis for the first permanent molars. The rejection of hypothesis H_01_, which posited no significant gender-based differences, revealed that boys are more prone to advanced carious lesions, emphasizing the need for gender-specific preventive strategies. Similarly, the rejection of hypothesis H_02_, which posited no significant differences between untreated and treated carious lesions, demonstrated critical gaps in the accessibility and utilization of preventive care, particularly dental sealants. Identifying these deficiencies underscores the urgent need to develop national oral health education and prevention programs, such as school-based dental hygiene campaigns and public awareness initiatives targeting both children and parents. Furthermore, increasing public funding for preventive treatments, such as sealants, and providing incentives for dental practitioners to deliver care in underserved areas are critical in improving access and equity. Targeted interventions addressing these systemic barriers and disparities are essential in mitigating the burden of oral disease and improving long-term dental outcomes for children. Implementing these measures could significantly reduce the burden of oral disease and improve dental health outcomes for children at the national level.

## Figures and Tables

**Table 1:** International Caries Detection and Assessment System.

code 0	Sound tooth surface; no evidence of caries after prolonged air drying (5 s)
code 1	First visual change in enamel: opacity or discoloration (white or brown) is visible at the entrance to the pit or fissure after prolonged air drying, which is not or hardly seen on wet surface
code 2	Distinct visual change in enamel: opacity or discoloration distinctly visible at the entrance to the pit and fissure when wet; lesion must still be visible when dry
code 3	Localized enamel breakdown due to caries with no visible dentine or underlying shadow: opacity or discoloration wider than the natural fissure/fossa when wet and after prolonged air drying
code 4	Underlying dark shadow from dentine with or without localized enamel breakdown
code 5	Distinct cavity with visible dentine: visual evidence of demineralization and dentine exposed
code 6	Extensive cavitation, with more than half of the surface affected

**Table 2:** Dental status of the first permanent molars.

Status	Total (n = 2064)	Girls (n = 1176)	Boys (n = 888)	Percentage (Total)	Percentage (Girls)	Percentage (Boys)
Extracted, maxillary	0	0	0	0.00%	0.00%	0.00%
Extracted, mandibular	15	3	12	0.73%	0.26%	1.35%
Sealed, maxillary	72	45	27	3.49%	3.83%	3.04%
Sealed, mandibular	57	42	15	2.76%	3.57%	1.69%
Filled, maxillary	84	57	27	4.07%	4.85%	3.04%
Filled, mandibular	108	72	36	5.23%	6.12%	4.05%
ICDAS 0, maxillary	255	123	132	12.35%	10.46%	14.86%
ICDAS 0, mandibular	150	63	87	7.27%	5.36%	9.80%
ICDAS 1–2, maxillary	150	96	54	7.27%	8.16%	6.08%
ICDAS 1–2, mandibular	168	93	75	8.14%	7.91%	8.45%
ICDAS 3, maxillary	147	108	39	7.12%	9.18%	4.39%
ICDAS 3, mandibular	192	135	57	9.30%	11.48%	6.42%
ICDAS 4–6, maxillary	387	186	201	18.75%	15.82%	22.64%
ICDAS 4–6, mandibular	279	153	126	13.52%	13.01%	14.19%

**Table 3:** Distribution of carious lesions by type.

Type of Lesions	Percentage
Located on occlusal surfaces or associated with vestibular or palatal pits	87.46%
On proximal surfaces	8.16%
Atypical lesions	2.28%
Root remnants	2.08%

## Data Availability

All data regarding this manuscript can be checked with corresponding author.

## References

[1] World Health Organization (2022). Global Oral Health Status Report: Towards Universal Health Coverage for Oral Health by 2030.

[2] Bratu E. (2005). Practica Pedodontică.

[3] Alfuriji S., Alamro H., Kentab J., Alosail L., Alali L., Altuwaijri N., Alalwan R. (2023). Ectopic Permanent Molars: A Review. Dent. J..

[4] Stoica S.N., Nimigean V., Vîrlan M.J.R., Nimigean V.R. (2023). The Pathology of the First Permanent Molar during the Mixed Dentition Stage—Review. Appl. Sci..

[5] Saber A.M., Altoukhi D.H., Horaib M.F., El-Housseiny A.A., Alamoudi N.M., Sabbagh H.J. (2018). Consequences of early extraction of compromised first permanent molar: A systematic review. BMC Oral Health.

[6] Hong H., Zhou J., Fan Q., Jiao R., Kuang Q., Zhou H., Hua C., Yang Z., Lai W., Long H. (2023). Characteristics of Spatial Changes in Molars and Alveolar Bone Resorption among Patients with Loss of Mandibular First Molars: A CBCT-Based Morphometric Study. J. Clin. Med..

[7] Chen Y.C., Chen C.Y.-H., Chen M.-C., Ko E.W.-C., Lin C.-H. (2023). Dental Occlusion Characteristics for Treatment Decision-Making Regarding Surgery-First Approach in Orthodontics. J. Clin. Med..

[8] Rajashekhara B.S., Keyur J.M., Bhavna D., Poonacha K.S. (2012). Management of early loss of first permanent molar: A new technique. J. Indian Soc. Pedod. Prev. Dent..

[9] Carvalho J.C. (2014). Caries process on occlusal surfaces: Evolving evidence and understanding. Caries Res..

[10] Moca A.E., Vaida L.L., Negruțiu B.M., Moca R.T., Todor B.I. (2021). The Influence of Age on the Development of Dental Caries in Children. A Radiographic Study. J. Clin. Med..

[11] Wang Z., Rong W., Xu T. (2022). Effect of Fluoride Varnish in Preventing Dental Caries of First Permanent Molars: A 24-Month Cluster Randomized Controlled Trial. Int. J. Environ. Res. Public Health.

[12] Torlińska-Walkowiak N., Łukaszewicz K., Morawska A., Sowińska A., Pawlaczyk-Kamieńska T., Opydo-Szymaczek J. (2023). Diet, Oral Hygiene Habits, and Approach to Dental Visits of Early School-Aged Children during the COVID-19 Pandemic and Possible Long-Term Health Consequences. J. Clin. Med..

[13] Sava-Rosianu R., Campus G., Matichescu A., Balean O., Dumitrache M.A., Lucaciu P.O., Daguci L., Barlean M.C., Mari-cutoiu L., Postolache M. (2021). Caries Prevalence Associated with Oral Health-Related Behaviors among Romanian Schoolchildren. Int. J. Environ. Res. Public Health.

[14] Dumitrescu R., Sava-Rosianu R., Jumanca D., Galuscan A. (2022). Dental Caries, Oral Health Behavior, and Living Conditions in 6–8-Year-Old Romanian School Children. Children.

[15] Rebelo M.A.B., Rebelo Vieira J.M., Pereira J.V., Quadros L.N., Vettore M.V. (2018). Does Oral Health Influence School Performance and School Attendance? A Systematic Review and Meta-Analysis. Int. J. Paediatr. Dent..

[16] Righolt A.J., Jevdjevic M., Marcenes W., Listl S. (2018). Global-, Regional-, and Country-Level Economic Impacts of Dental Diseases in 2015. J. Dent. Res..

[17] Chaves J.C., Santos T.R., Marsillac M.W.S., Alexandria A., Fidalgo T.K.S. (2021). Assessment of dental caries and intervention in the first permanent molars of Brazilian children. Pesqui. Bras. Odontopediatria Clín. Integr..

[18] Nazir M.A., Bakhurji E.A., Gaffar B.O., Al-Khalifa K. (2019). Permanent Molar Caries and its Association with Carious Lesions in Other Permanent Teeth. J. Clin. Diagn. Res..

[19] Kazeminia M., Abdi A., Shohaimi S., Jalali R., Vaisi-Raygani A., Salari N., Mohammadi M. (2020). Dental caries in primary and permanent teeth in children’s worldwide, 1995 to 2019: A systematic review and meta-analysis. Head Face Med..

[20] Zhu F., Chen Y., Yu Y., Xie Y., Zhu H., Wang H. (2021). Caries Prevalence of the First Permanent Molars in 6–8 Years Old Children. PLoS ONE.

[21] Yousaf M., Aslam T., Saeed S., Sarfraz A., Sarfraz Z., Cherrez-Ojeda I. (2022). Individual, Family, and Socioeconomic Contributors to Dental Caries in Children from Low- and Middle-Income Countries. Int. J. Environ. Res. Public Health.

[22] Mačiulskienė V., Razmienė J., Andruškevičienė V., Bendoraitienė E. (2020). Estimation of Caries Treatment Needs in First Permanent Molars of Lithuanian 5–6-Year-Old Children, Based on Caries Lesion Activity Assessment. Medicina.

[23] Tanase M., Stanciu I.-A., Zmarandache D., Feraru V. (2018). Study on Caries Prevalence and Clinical Performance of Restorations Applied on the First Permanent Molar. Rom. J. Stomatol..

[24] International Caries Detection and Assessment System (ICDAS) Coordinating Committee https://www.iccms-web.com/uploads/asset/592848be55d87564970232.pdf.

[25] Mazur M., Corridore D., Ndokaj A., Ardan R., Vozza I., Babajko S., Jedeon K. (2023). MIH and Dental Caries in Children: A Systematic Review and Meta-Analysis. Healthcare.

[26] Urvasizoglu G., Bas A., Sarac F., Celikel P., Sengul F., Derelioglu S. (2023). Assessment of Permanent First Molars in Children Aged 7 to 10 Years Old. Children.

[27] Szöke J., Petersen P.E. (2020). Changing Levels of Dental Caries over 30 Years among Children in a Country of Central and Eastern Europe—The Case of Hungary. Oral Health Prev. Dent..

[28] Lešić S., Dukić W., Kriste Z.Š., Tomičić V., Kadić S. (2019). Caries prevalence among schoolchildren in urban and rural Croatia. Cent. Eur. J. Public Health.

[29] Vrbič V., Vrbič M. (2016). Epidemiology of Caries in 12-Year-Olds in Slovenia 1987–2013. Oral Health Prev. Dent..

[30] Petersen P.E., Danila I., Delean A., Grivu O., Ionita G., Pop M., Samolia A. (1994). Oral Health Status among Schoolchildren in Romania, 1992. Community Dent. Oral Epidemiol..

[31] Baciu D., Danila I., Balcos C., Gallagher J.E., Bernabé E. (2015). Caries experience among Romanian schoolchildren: Prevalence and trends 1992–2011. Community Dent. Health.

[32] Sfeatcu R., Cărămidă M., Sava-Rosianu R., Lupșa Matichescu M., Galuscan A., Dumitrache M.A. (2023). Carious Status and Socio-Behavioral Risk Factors among 12-Year-Old Children in South-Central Region of Romania. BMC Oral Health.

[33] Gavrilescu A., Petrescu A.-M., Dumitriu H.-T. (2020). Oral Health Status among Children in Romania. Rom. J. Oral Rehabil..

[34] Tudoroniu C., Popa M., Iacob S.M., Pop A.L., Năsui B.A. (2020). Correlation of Caries Prevalence, Oral Health Behavior and Sweets Nutritional Habits among 10 to 19-Year-Old Cluj-Napoca Romanian Adolescents. Int. J. Environ. Res. Public Health.

[35] Cvikl B., Moritz A., Bekes K. (2018). Pit and Fissure Sealants—A Comprehensive Review. Dent. J..

[36] Cabalén M.B., Molina G.F., Bono A., Burrow M.F. (2022). Nonrestorative Caries Treatment: A Systematic Review Update. Int. Dent. J..

[37] Bereșescu L., Păcurar M., Bica C.I., Vlasa A., Stoica O.E., Dako T., Petcu B., Esian D. (2022). The Assessment of Sealants’ Effectiveness in Arresting Non-Cavitated Caries Lesion—A 24-Month Follow-Up. Healthcare.

[38] Lam P.P., Sardana D., Lo E.C., Yiu C.K. (2021). Fissure sealant in a nutshell. Evidence-based meta-evaluation of sealant’ effectiveness in caries prevention and arrest. J. Evid. Based Dent..

[39] Ahovuo-Saloranta A., Forss H., Walsh T., Nordblad A., Mäkelä M., Worthington H.V. (2017). Pit and fissure sealants for preventing dental decay in permanent teeth. Cochrane Database Syst. Rev..

[40] Kühnisch J., Bedir A., Lo Y.F., Kessler A., Lang T., Mansmann U., Heinrich-Weltzien R., Hickel R. (2020). Meta-analysis of the longevity of commonly used pit and fissure sealant materials. Dent. Mater..

[41] Bromo F., Guida A., Santoro G., Peciarolo M.R., Eramo S. (2011). Pit and Fissure Sealants: Review of Literature and Application Technique. Minerva Stomatol..

[42] Gavrilă-Ardelean L., Gavrilă-Ardelean M., Lackner A.K., Kozma A. (2021). The role and resources of fluor in oral health. ORL.

[43] Valenti C.S., Bozza S., Pagano Ciurnella E., Lomurno G., Capobianco B., Coniglio M., Cianetti S., Marinucci L. (2021). Use of the Er:YAG Laser in Conservative Dentistry: Evaluation of the Microbial Population in Carious Lesions. Materials.

[44] Kassebaum N.J., Bernabé E., Dahiya M., Bhandari B., Murray C.J., Marcenes W. (2015). Global burden of untreated caries: A systematic review and metaregression. J. Dent. Res..

[45] Abramovitz I., Zini A., Kessler Baruch O., Kedem R., Protter N.E., Shay B., Yavnai N., Zur D., Mijiritsky E., Almoznino G. (2021). SOS teeth with advanced caries and sociodemographic indicators, health-related habits and dental attendance patterns: Data from the Dental, Oral, Medical Epidemiological (DOME) nationwide records-based study. BMC Oral Health.

[46] Petersen P.E. (2003). The World Oral Health Report 2003: Continuous improvement of oral health in the 21st century--the approach of the WHO Global Oral Health Programme. Community Dent. Oral Epidemiol..

[47] Ionaș M. (2017). Dental Care Awareness among Mothers of Children from Disadvantaged Families in Sibiu County. Acta Medica Transilv..

[48] Stepurko T., Pavlova M., Groot W. (2016). Overall satisfaction of health care users with the quality of and access to health care services: A cross-sectional study in six Central and Eastern European countries. BMC Health Serv. Res..

[49] Karnaki P., Katsas K., Diamantis D.V., Riza E., Rosen M.S., Antoniadou M., Gil-Salmerón A., Grabovac I., Linou A. (2022). Dental Health, Caries Perception and Sense of Discrimination among Migrants and Refugees in Europe: Results from the Mig-HealthCare Project. Appl. Sci..

[50] Ghanbarzadegan A., Balasubramanian M., Luzzi L., Brennan D., Bastani P. (2021). Inequality in dental services: A scoping review on the role of access toward achieving universal health coverage in oral health. BMC Oral Health.

[51] Luan Y., Sardana D., Jivraj A., Liu D., Abeyweera N., Zhao Y., Cellini J., Bass M., Wang J., Lu X. (2024). Universal coverage for oral health care in 27 low-income countries: A scoping review. glob health res policy. Glob. Health Res. Policy.

